# Gullies and Moraines Are Islands of Biodiversity in an Arid, Mountain Landscape, Asgard Range, Antarctica

**DOI:** 10.3389/fmicb.2021.654135

**Published:** 2021-06-10

**Authors:** Adam J. Solon, Claire Mastrangelo, Lara Vimercati, Pacifica Sommers, John L. Darcy, Eli M. S. Gendron, Dorota L. Porazinska, S. K. Schmidt

**Affiliations:** ^1^Department of Ecology and Evolutionary Biology, University of Colorado, Boulder, Boulder, CO, United States; ^2^School of Public Health, University of California, Berkeley, Berkeley, CA, United States; ^3^Division of Biomedical Informatics and Personalized Medicine, University of Colorado-Anschutz Medical Campus, Denver, CO, United States; ^4^Department of Entomology and Nematology, University of Florida, Gainesville, FL, United States

**Keywords:** cold deserts, gullies, microbial oases, extremophiles, biological soil crusts, *Bryum*, cryobiosphere

## Abstract

Cold, dry, and nutrient-poor, the McMurdo Dry Valleys of Antarctica are among the most extreme terrestrial environments on Earth. Numerous studies have described microbial communities of low elevation soils and streams below glaciers, while less is known about microbial communities in higher elevation soils above glaciers. We characterized microbial life in four landscape features (habitats) of a mountain in Taylor Valley. These habitats varied significantly in soil moisture and include moist soils of a (1) lateral glacial moraine, (2) gully that terminates at the moraine, and very dry soils on (3) a southeastern slope and (4) dry sites near the gully. Using rRNA gene PCR amplicon sequencing of Bacteria and Archaea (16S SSU) and eukaryotes (18S SSU), we found that all habitat types harbored significantly different bacterial and eukaryotic communities and that these differences were most apparent when comparing habitats that had macroscopically visible soil crusts (gully and moraine) to habitats with no visible crusts (near gully and slope). These differences were driven by a relative predominance of Actinobacteria and a *Colpodella* sp. in non-crust habitats, and by phototrophic bacteria and eukaryotes (e.g., a moss) and predators (e.g., tardigrades) in habitats with biological soil crusts (gully and moraine). The gully and moraine also had significantly higher 16S and 18S ESV richness than the other two habitat types. We further found that many of the phototrophic bacteria and eukaryotes of the gully and moraine share high sequence identity with phototrophs from moist and wet areas elsewhere in the Dry Valleys and other cold desert ecosystems. These include a Moss (*Bryum* sp.), several algae (e.g., a *Chlorococcum* sp.) and cyanobacteria (e.g., *Nostoc* and *Phormidium* spp.). Overall, the results reported here broaden the diversity of habitat types that have been studied in the Dry Valleys of Antarctica and suggest future avenues of research to more definitively understand the biogeography and factors controlling microbial diversity in this unique ecosystem.

## Introduction

In the cold, nutrient-poor, and hyper-arid McMurdo Dry Valleys (MDV) of Antarctica, water availability serves as a primary factor governing the persistence and distribution of life (Kennedy, 1993). Climate, generally understood as long-term records of air temperature and precipitation, determines water availability on a regional scale, whereas variations in terrain (e.g., slope, aspect, shade, and soil conditions) determine water availability on a local level. Evaluating how differences in mountain terrain (habitats) influence microbial community structure and diversity in the MDV will provide a better understanding of microbial biogeography and connectivity between mountain slopes and the glaciers, lakes, and soils in valleys below.

Within the MDV, there are three geomorphic zones that are determined by interactions between climate conditions and landforms. These zones are defined by summertime mean air temperature and relative humidity as well as prevailing winds and precipitation and include the coastal thaw zone (CTZ), the inland mixed zone (IMZ), and the stable upland zone (SUZ) (Fountain et al., 2014). These zones correspond with elevation above sea level and distance from the coast. For example, in Taylor Valley, the warmest and wettest zone, the CTZ, is found dozens of kilometers from the coast at low elevations and the colder and drier IMZ is found a few kilometers from the coast at higher elevations. The SUZ, the coldest and driest zone, occurs in the highest elevations and valleys farthest from the coast. In addition, the zones correspond with differences in geologic histories (Bockheim et al., 2008), parent material and stages of weathering (Hall et al., 2000; Bockheim, 2002), and soil chemistries (Bockheim, 1997). These four factors create physical environments distinct from one another that govern moisture availability (Campbell et al., 1998). Previous work at lower elevations in the MDV indicates water is a primary limiting factor for life (Gooseff et al., 2003; Lee et al., 2018) and there is evidence of a lower soil moisture limit for eukaryotic phototrophy (Fell et al., 2006). Soil water content is also the primary factor controlling other microbial functions (Barrett et al., 2006; Ball et al., 2009; Niederberger et al., 2019) and moss photosynthesis in the MDV (Pannewitz et al., 2005). On a broader scale soil moisture affects biological soil crust formation in other cold deserts (Costello et al., 2009; Solon et al., 2018), and even in hot deserts like the Negev Desert (Kidron and Benenson, 2014; Hagemann et al., 2017).

While numerous studies have described microbial communities of the CTZ (Van Horn et al., 2016; Feeser et al., 2018) and SUZ (Wood et al., 2008; Goordial et al., 2016), less is known of microbes in the intermediate elevation sites of the IMZ. Existing studies that include life from the IMZ focus exclusively on bacterial endoliths (Archer et al., 2017), soil yeasts (Connell et al., 2008), or micro-eukaryotes (Fell et al., 2006), or include a single terrain feature (Smith et al., 2006; Lee et al., 2012). One mountain chain within the Transantarctic, the Asgard Range, establishes the northern border of Taylor Valley and is representative of the IMZ in the valley. As part of a larger study of factors contributing to the assembly of microbial communities in cryoconite holes on Canada Glacier and other glaciers in Taylor Valley (cf. Sommers et al.,2019a,b,c), the four mountain habitats discussed in the present study were identified as possible sources of inoculum for the cryoconite holes found on Canada Glacier below.

To our knowledge no previous studies have sampled all domains of soil biota across a range of habitats found within one mountain landscape of the IMZ of Taylor Valley. We identified four habitats with different gravimetric soil moisture levels: moister soils of a (1) lateral glacial moraine (henceforth, moraine), (2) gully (gully), and drier soils of a (3) southeastern slope (slope) and (4) alongside the gully (near gully). Our study addresses the following questions: How different are microbial communities among these habitats? Do the habitats with higher soil moisture support more diverse communities? Are phototrophs of the IMZ also found in lower elevations of the MDV or other cold deserts? By incorporating the full spectrum of life found in each habitat (Bacteria, Archaea, and eukaryotes) as well as identifying habitat-specific community compositions and individual organisms most associated with those habitats, we used a natural experiment to explore how the variation of terrain within a geomorphic zone governs soil moisture and influences microbial community structure in the IMZ of the MDV and identified potential connections with microbial communities of cryoconite holes on the glaciers below.

## Materials and Methods

### Site Description

The McMurdo Dry Valleys (MDV) are ∼4,800 km^2^ of mostly ice-free expanse and are the largest ice-free area of Antarctica. Located in Southern Victoria Land between the East Antarctica Ice Sheet and the Ross Sea, this cold desert environment is the product of multiple physical factors, one of which is the presence of the Transantarctic Mountains which extend across most of the continent and divide the East and West Antarctica Ice Sheets. One of these ranges, the Asgard, separates Taylor Valley from Wright Valley to the north. Taylor Valley was first explored during the 1907 British Antarctic Expedition and in recent decades has seen an enhanced focus of scientific investigation since the establishment of the McMurdo Long-Term Ecological Research (LTER) site in 1993.

**FIGURE 1:**
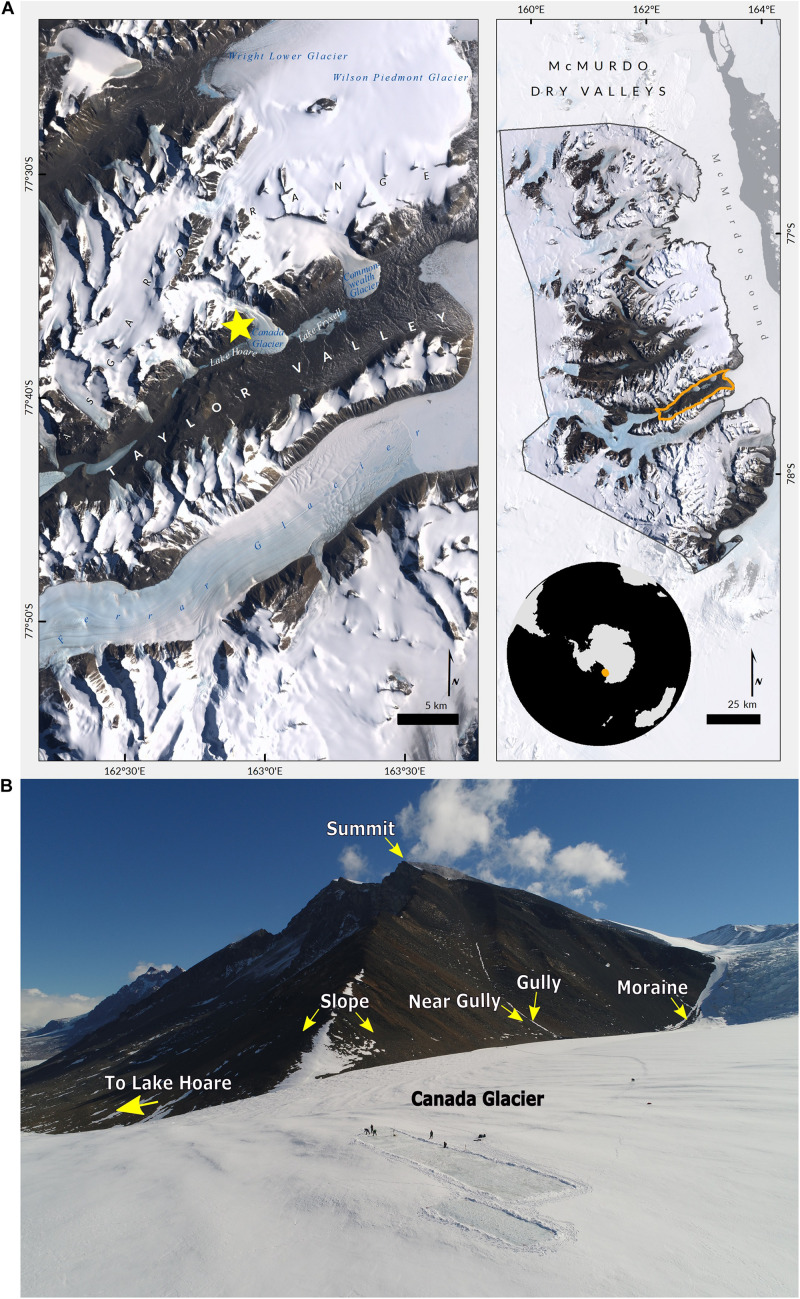
Research site. **(A)** Satellite map of the McMurdo Dry Valleys (right) and Taylor Valley Valley (left). The yellow star identifies the location of the area sampled within the Asgard Range (images: Cathleen Torres Parisian, Polar Geospatial Center). **(B)** View from drone taken above Canada Glacier with arrows pointing to the habitats sampled in this study, and other key landmarks (photo: Brendan Hodge, UNAVCO).

Of the three geomorphic zones in the MDV most studies in Taylor Valley take place in the lower elevation coastal thaw zone (CTZ), defined by mean summer air temperatures > −5°C and a wet-active layer of permafrost. However, the higher elevations in the valley, in the Asgard Mountains and Kukri Hills, which form the southern edge of Taylor Valley, exist in the inland mixed zone (IMZ). The IMZ is defined by an isotherm of mean summer air temperature between −5 and −10°C and a dry active layer (Fountain et al., 2014). For this study we selected a mountain in the Asgard Range that rises above the Lake Hoare field camp and Canada Glacier- 77°36′36.71″ S, 162°50′07.32″ E, 1,180 m a.s.l (Figure 1).

The first habitat is representative of most of the Asgard mountains and is located on the rocky, barren, southeastern slope, which we refer to as “slope.” The second and third habitats are defined by inside and 10m outside of a gully that runs vertically down the mountain’s east side, which we, respectively, call “gully” and “near gully.” The fourth habitat is a lateral moraine that runs along the western edge of the glacier and eastern slope of the mountain, which we term “moraine.” It is noteworthy that the gully runs perpendicular to the moraine and intersects it. Each habitat offered a different combination of terrain- slope, aspect, and other geologic and geographic features- but we were especially interested in the differences of gravimetric soil moisture that are present in this hyper-arid mountain landscape. While gravimetric soil moisture is limited to the amount of water at a single point in time, and not a full indicator of water availability, this metric has been used in previous studies of life in the dry valleys (Barrett et al., 2006; Ball et al., 2009; Niederberger et al., 2019). Additionally, biological soil crusts were seen in the gully and moraine and are referred to as crust habitats, whereas no soil crusts were observed in the near gully and slope and are referred to as non-crust habitats. Photos of habitats and additional sample information are included as supplemental materials (Supplementary Material 1 and Supplementary Table 1).

### Soil Collection

Mineral soils were collected over two field seasons using sterile technique with an ethanol-cleaned stainless-steel spoon from the top 0 to 4 cm of the surface and homogenized in individual, sterile Whirl-Pak^®^ bags. The slope was sampled on 24 Dec 2016 in a lateral transect from 77° 36′ 35.6394″ S, 162° 55′ 29.892″ E (287 m a.s.l.) to 77° 36′ 43.272″ S, 162° 55′ 36.336″ E (256 m a.sl.) for a total of eight samples. The gully and near gully were sampled on 22 Jan 2018 along an elevational gradient from 77° 36′ 29.628″ S, 162° 54′ 22.2012″ E (424 m a.s.l) to 77° 36′ 26.6394″ S, 162° 54′ 48.8412″ E (358 m a.s.l). Samples were collected from both inside the gully, where green and black crusts were occasionally visible, and a corresponding sample 10 m outside the gully for a total twenty-one gully and nine near gully samples. The nine moraine samples were collected on 25 Jan 2018 along an elevational gradient from 77°36′13.50″ S, 162°54′14.26″ E (459 m a.s.l) to 77°36′18.86″ S, 162°54′33.57″ E (402 m a.s.l.) and featured occasional visible crusts. MDV environmental regulations were followed and influenced the locations and limited amounts of sampling. Soil temperatures were recorded in the gully and moraine from 26 November 2018 to 24 January 2019 with HOBO Pendant^®^ Temperature/Light data loggers. Loggers were placed within a few mm of the surface along elevational gradients with 8 loggers in the gully and 4 loggers in the moraine.

### Sample Processing, DNA Sequencing, and Bioinformatics

All soils were transported down the mountain and kept frozen in −20°C freezers located at Lake Hoare camp, then flown in coolers to the A.P. Crary Science and Engineering Center at McMurdo Station where they were kept at −70°C. At the end of each season, the samples were shipped at −20°C to Boulder, CO, United States and stored at −70°C until further processing took place. Gravimetric soil moisture was determined by weighing 3 g of soil (wet weight), drying at 100°C for 24 h, and re-weighing the mass again (dry weight). The difference between the two weights was then divided by the dry weight for soil moisture (%). Gravimetric soil moisture determines the water content of soil, although not the overall availability of it seasonally nor at the time of sampling, and has been used as a metric for soil microbial studies in the MDV (Barrett et al., 2006; Fell et al., 2006; Ball et al., 2009; Niederberger et al., 2019). Microinveterbrate counts were conducted following protocols outlined in Porazinska et al. (2018) but which included homogenizing and subsetting soil and storing at 4°C before sieving and counting. DNA was extracted from 0.3 to 0.45 g/soil using a Qiagen PowerSoil DNA Extraction kits (Qiagen, Hilden, Germany) and the concentration of DNA was quantified with a Qubit fluorometer (Invitrogen Corp., CA, United States). Qubit values in ng/μL were back calculated into ng/g soil. DNA was then amplified in triplicates with Earth Microbiome Project primers for the 16S SSU rRNA gene (515f-806r) and 18S SSU rRNA gene (1391f-EukBr), amplified triplicates pooled and normalized to equimolar concentrations with a SequalPrep Normalization Plate Kit (Invitrogen Corp., CA, United States). Pooled samples were then sequenced at University of Colorado-Boulder BioFrontiers Sequencing Facility on an Illumina MiSeq 2 × 250 bp for 16S SSU amplicons and 2 × 150 bp for 18S SSU amplicons. Raw sequence reads are stored in the NCBI SRA database under BioProject accession number PRJNA721735. All bioinformatics were conducted with either PYTHON (version 2.7) or R (version 3.6.1) programming languages in R Studio (version 1.2.1335). Additional labels and image grouping for figures were conducted in Inkscape^1^. Samples were processed with a template developed by Angela Oliveira and Hannah Holland-Moritz^2^ was modified from the DADA2 tutorial pipeline^3^. This template provides a pipeline where reads are demultiplexed and primers are removed (idemp; cutadapt, version 1.18) (Martin, 2011). Following DADA2 protocols any sequences with an “N,” that is undetermined base pairs, were filtered out. Using a graph produced in the DADA2 pipeline of the frequency of each quality score at each base position sequence lengths were trimmed when the average PHRED score dropped below 30. Error rate learning for DADA2 was set to the default minimum number of total bases to use for error rate learning at 1e8. After trimming and filtering exact sequence variants (ESVs) were inferred, forward and reverse reads paired, chimeras removed, and taxonomy assigned (“dada2” package, version 1.6.0; “plotly” package, version 4.9.0). Taxonomy was provided by the SILVA SSU 132 database for both 16S and 18S reads (Quast et al., 2013). For richness estimation samples were pooled before ESVs were inferred. Further processing with “phyloseq” R package (McMurdie and Holmes, 2013) included subsetting data for study-specific samples, removing erroneous domain assignments (e.g., eukaryotes in 16S data) and contamination (e.g., human DNA). Chloroplasts and mitochondria were also removed from the 16S. Finally, samples with very low read counts (<1,000) were filtered from the data set (“phyloseq” package, version 1.32.0). The 16S SSU dataset started with 725615 reads and the 18S SSU dataset 502052 reads. After processing the 16S contained 663595 filtered reads and 4340 ESVs across 44 samples and the 18S dataset 499200 filtered reads and 527 ESVs across 32 samples. For richness estimation, the pooled datasets contained 766311 16S reads and 475343 18S reads. After processing the 16S dataset yielded 699606 reads and 4327 ESVs across 44 samples and the 18S dataset 472888 reads and 521 ESVs across 32 samples.

### Beta Diversity

The use of DNA-seq data for abundance-related analyses is limited to relative abundances and necessitates data transformations for compositional analyses (Gloor and Reid, 2016; Gloor et al., 2017). To determine differences between communities we transformed the data into Aitchison distances with, first, a zero-replacement function (“zCompositions” package, version 1.3.3–1), and second, center-log ratio (clr) transformation (“compositions” package, version 1.40–3) (Gloor and Reid, 2016). Principal Components Analysis (PCA) ordinations were created (“CoDaSeq” package, version 0.99.6, “ggplot2” package). PERMANOVA was used to test differences among communities by habitat based on centroids of their Aitchison distances. To determine which organisms were most responsible for the differences in the crust and non-crust communities we used the “ALDEx2” package (version 1.20.0). ALDEx2 determines which ESVs are most differentially relatively abundant between two groups by using median center-log ratio values (Fernandes et al., 2013, 2014). We assigned groups based on the presence or absence of crusts in the habitat type. The gully and moraine had crusts and the near gully, and slope did not have crusts. This yielded positive values indicating greater association with the crust communities and negative values more associated with non-crust communities. Additionally, an effect size for each ESV was computed as the difference between two groups divided by the dispersion within each respective group, and significance values were determined with Benjamini-Hochberg corrected *P*-values of Wilcoxon tests. We only report those ESVs that had effect sizes greater than 1, or less than −1, and *p* < 0.05.

### Alpha Diversity

For a quantitative estimate of ESV richness we used the “breakaway” package (version 4.7.2) to generate estimates with “breakaway” (Willis and Bunge, 2015) and conduct null hypothesis significance testing with “betta” (Willis et al., 2017). Two sample outliers from the 16S dataset (GB7 and SL5) were removed as those samples provided estimate ranges with negative numbers of ESVs which is not biologically possible (Supplementary Material 2). To assess dominant organisms in each habitat, we produced boxplots with the top 10 average ESVs (median) of each habitat using untransformed relative abundances.

## Results

### Site Characteristics

**FIGURE 2:**
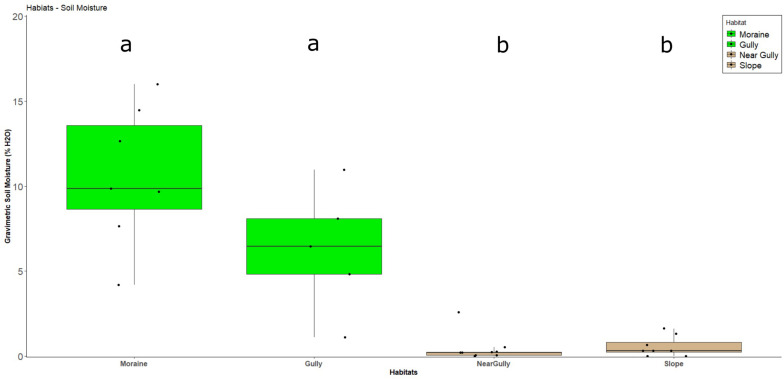
Soil moisture boxplots of gravimetric soil moisture by habitat. Green identifies habitats with crusts. Non-crust habitats are in tan. Each dot is a single sample, the box represents the 25–75% quartile values, and the dark line in the box represents median values of soil moisture. Lower-case letters indicate the presence of statistical difference among habitats as determined by pairwise Dunn tests.

The most obvious macro-scale differences between the sites we sampled are that the gulley and moraine are areas that accumulate wind-blown snowpack due to local topography (Figure 1B and see Supplementary Figures 1, 3, 4). It is likely that the residual water from this snowpack accumulation accounted for the significantly higher soil moisture levels observed in those two habitats at the time of sampling (Dunn Test: *p* < 0.02) (Table 1 and Figure 2). Likewise, levels of estimated biomass (DNA concentration) and direct microscopic counts of tardigrades and rotifers (but not nematodes) were generally higher in the gulley and on the moraine compared to the drier sites sampled nearby (Table 2).

**TABLE 1:** Environmental data by habitat.

Habitat	Elevation^a^	Aspect	Snowbank formation^b^	Soil moisture^c^	Soil temperature^d^
Moraine	407–457	South	Yes	4.18–15.99	Nov (−8.58, 16.5) Dec (−3.85, 17.1) Jan (−2.24, 17.4)
Gully	320–441	East	Yes	1.10–10.96	Nov (−7.66, 27.0) Dec (−4.19, 29.5) Jan (−4.02, 28.6)
Near gully	320–441	East	No	0.00–2.59	n/a
Slope	256–287	East/South	No	0.00–1.62	n/a

**^*a*^Meters above sea level. ^*b*^Does snowbank accumulation occur over the summer? ^*c*^Gravimetric soil moisture (%) values across samples. ^*d*^Mean minimum and maximum temperatures in degrees Celsius (Nov = Nov 26–30, Dec = Dec 1–31, Jan = Jan 1–24).**

**TABLE 2:** Abundance data by habitat.

Habitat	Biocrust	Biomass^a^	Rotifers^b^	Tardigrades^c^	Nematodes^d^
Moraine	Yes	928–14,200	0–7	3–123	0–364
Gully	Yes	3690–400,000 +	0–2	8–55	0–10
Near gully	No	1,370–3,060	0–1	0	7–44
Slope	No	103–1,690	n/a	n/a	n/a

**^*a*^Biomass in ng DNA/g of soil (Qubit Fluorometer). ^*b*^Number of Rotifers, alive or dead, standardized to 20 g dry weight soil. ^*c*^Number of Tardigrades, alive or dead, standardized to 20 g dry weight soil. ^*d*^Number of Nematodes, alive or dead, standardized to 20 g dry weight soil.**

### Communities

**FIGURE 3:**
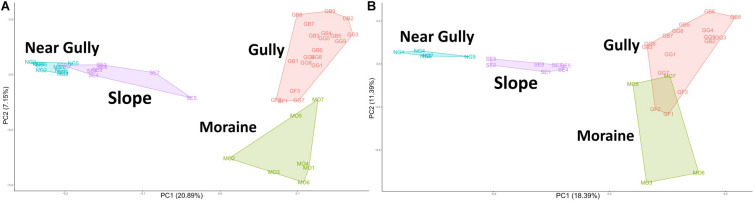
Principal components analysis (PCA) plot of Aitchison distances with polygons capturing all samples from within a habitat. **(A)** 16S, **(B)** 18S. Pairwise PERMANOVA significance test revealed the centroid of each habitat as significantly different from all other habitats for both the bacterial and eukaryotic communities (16S *p* = 0.001; 18S *p* < 0.006).

PCA and PERMANOVA analyses showed significant clustering of communities by habitat type for both 16S and 18S communities (Figure 3). The communities in the habitats that contain spatially irregular biological soil crusts (gully and moraine) separated along the *x*-axis from communities that had no visible soil crusts (near gully and slope). These separations were significant for both the 16S (PERMANOVA: *R*^2^ = 0.28, *p* = 0.001) and 18S (*R*^2^ = 0.29, *p* = 0.001) communities, with *post hoc* pairwise tests confirming significant differences between every habitat pairing (Pairwise PERMANOVA, FDR-adjusted: 16S *p* = 0.001, 18S *p* < 0.006).

**FIGURE 4:**
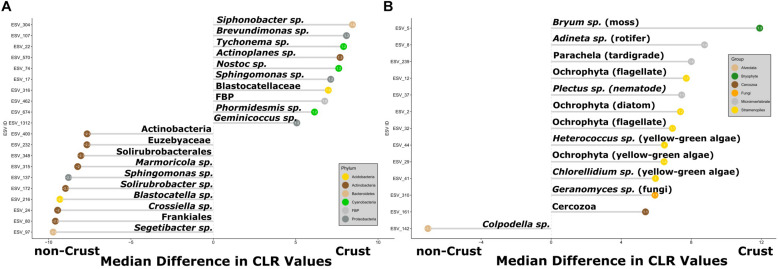
Organisms with the greatest differential relative abundances between samples from crust communities and those from non-crust communities **(A)** 16S, and **(B)** 18S. The *x*-axis displays the difference in median center-log ratio (CLR) transformed abundances and only ESVs with effect sizes > 1 or < –1 and *p*-values < 0.05 are displayed. The value inside the dot is the effect size. The positive values indicate ESVs in crusts and negative values indicate ESVs in non-crusts. Each ESV is labeled with the lowest taxonomic assignment from the SILVA 132 or NCBI GenBank databases. The colored dots refer to bacterial phyla or higher-level taxonomic grouping for Eukaryotes.

Differential relative abundance of ESVs (Fernandes et al., 2014) differed significantly between crust and non-crust communities for both 16S and 18S (Figure 4) (Wilcoxon, BH-adjusted: 16S *p* < 0.0001, 18S *p* < 0.01). For the 16S communities, most of the ESVs with greater differential abundance in the non-crust communities were Actinobacteria (7 out of 10 phylotypes), while the crust communities contained a wider diversity of phylotypes, including only 1 Actinobacteria ESV and notably 3 cyanobacteria ESVs among them (Figure 4A). The 18S analysis revealed the non-crust communities featured only a single member with higher differential abundance, an Alveolata (*Colpodella* sp.), while the crust communities had many more differentially abundant taxa including a moss (*Bryum* sp.) and higher order predators including a tardigrade, a nematode (*Plectus* sp.), and a rotifer (*Adineta* sp.) (Figure 4B).

**FIGURE 5:**
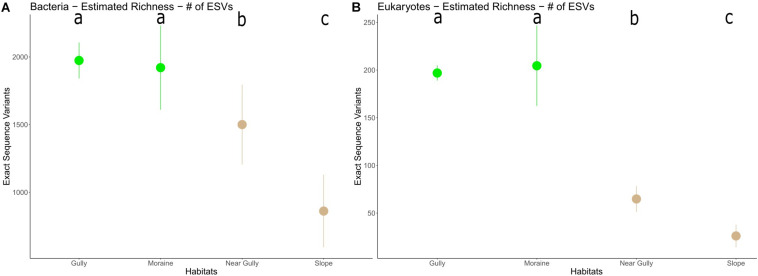
Microbial ESV richness in each habitat **(A)** 16S and **(B)** 18S. The dots represent the mean richness in each habitat with error bars indicating 95% CI. Lower case letters indicate significant differences among habitats (*p* < 0.003).

Richness estimates revealed higher levels of richness in the habitats with prominent soil crusts (gully and moraine) than in the habitats without soil crusts (near gully and slope) for both 16S (*p* < 0.003) and 18S (*p* < 0.001) communities (Figure 5). Also, among the non-crust habitats the near gully had significantly greater richness than the slope for both 16S (*p* < 0.001) and 18S (*p* < 0.001) communities.

### Organisms

**FIGURE 6:**
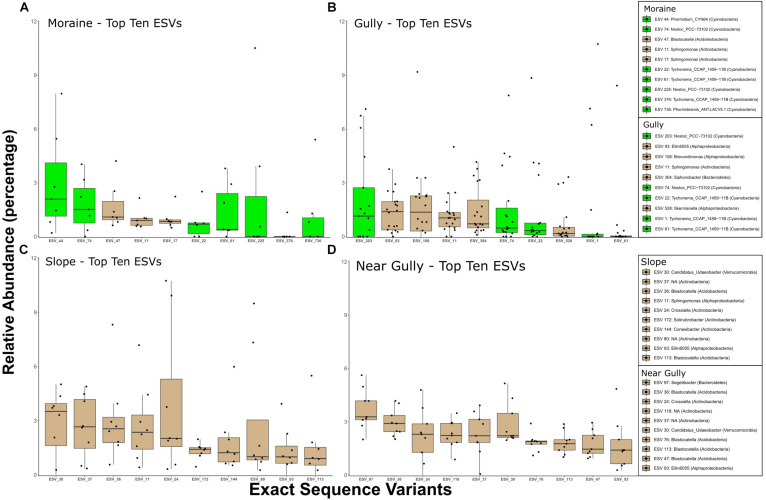
Top 10 16S ESVs from each habitat. **(A)** moraine, **(B)** gully, **(C)** slope, and **(D)** near gully. Boxplots are ordered by the median relative abundance of untransformed DNA-seq data and colored according to inferred energy-carbon acquisition-photoautotrophs (green) and chemoheterotrophs (tan).

**FIGURE 7:**
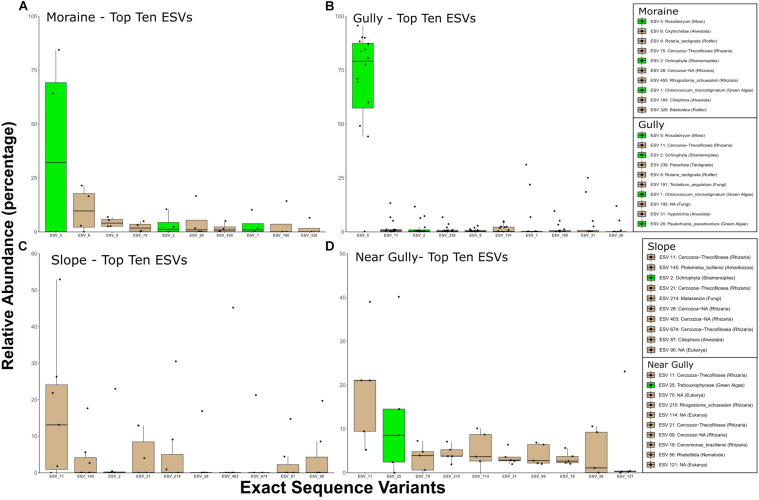
Top 10 18S ESVs from each habitat **(A)** moraine, **(B)** gully, **(C)** slope, and **(D)** near gully. Boxplots are ordered by the median relative abundance of untransformed DNA-seq data and colored according to inferred energy-carbon acquisition- photoautotrophs (green) and chemoheterotrophs (tan).

The differences between crust and non-crust microbial communities were even more evident when we examined the predominant organisms across habitat types. The relative abundances of the top 10 16S ESVs indicated that 16S crust communities were dominated by phototrophic Bacteria, whereas the nearby drier habitats had no phototrophs among the top-10 phylotypes (Figure 6). The Cyanobacteria in crust habitats included ESVs that most closely matched *Nostoc*, *Phormidium*, *Tychonema*, and *Phormidesmis*. In contrast, although eukaryotic phototrophs were present in the top 10 ESVs in all habitats, they were less abundant in the drier habitats (Figure 7). The most abundant 18S ESV in crust habitats was a moss (*Bryum* sp.) that shares 100% sequence identity with a moss previously described from the Dry Valleys (Seppelt et al., 2010). In addition, two genera of green algae from the class Chlorophyceae were also present (*Chlorococcum* in the gully and moraine, and *Paulschulzia* in the gully).

## Discussion

### How Different Are Microbial Communities Among Habitats?

We characterized microbial communities from mineral soils of four landscape features (habitats) and found that all habitats supported significantly different microbial communities (16S, *p* = 0.001; 18S, *p* < 0.006), although there was a notable separation between moist habitats with biological soil crusts (gully and moraine) and dry habitats with no crusts (near gully and slope) (Figure 3). These findings highlight the extent of environmental heterogeneity across this mountain landscape. These habitats were only a few hundred meters apart sharing similar elevations, parent material, geologic history, and climate (Bockheim, 2002; Fountain et al., 2014) and yet differences in habitat influenced not only differences in soil moisture but more importantly for this study, the presence or absence of biological soil crusts. Similar findings have been noted in ice-free desert soils of the Larsemann Hills and Bunger Hills regions of the continent, were physiochemical factors (moisture and organic matter) and the presence of crusts/mats (e.g., phototrophs) influenced bacterial community structure (Kudinova et al., 2020).

Given that crust vs. non-crust communities significantly separated on the main PCA axis (Figure 3), we carried out a differential relative abundance analysis (Fernandes et al., 2014) to examine which organisms contributed the most to this separation. For Bacteria, a wide variety of Actinobacteria drove the separation of the non-crust, drier communities, whereas a mix of major bacterial groups (e.g., Cyanobacteria and Proteobacteria) drove the separation of the crust communities (Figure 4A). These findings agree with previous studies in Taylor Valley in that elevated soil moisture corresponds with higher abundance of Cyanobacteria and reduced soil moisture corresponds with higher abundance of Actinobacteria (Niederberger et al., 2015; Lee et al., 2018; Rego et al., 2019), although Buelow et al. (2016) found a different pattern. A similar general pattern was also observed elsewhere on the continent at high elevations in the Sør Rondane Mountains of Queen Maud Land (Tytgat et al., 2016). Likewise, higher soil moisture was correlated with higher rates of CO_2_ and N_2_ fixation in *Nostoc*-associated microbial mats in Miers Valley (Sohm et al., 2020). Also, the preponderance of Actinobacteria and the lack of Cyanobacteria in other cold-dry ecosystems outside of Antarctica is well known (e.g., An et al., 2013; Gupta et al., 2015). For example, at extreme high elevation (>6,000 m) sites in the Atacama Desert, Actinobacteria are dominant, and Cyanobacteria are undetectable except near sources of water (Costello et al., 2009; Solon et al., 2018).

The eukaryotic taxa contributing to the separation of crust and non-crust communities were taxa associated with the crust habitats including a common moss (a *Bryum* species), a few Xanthophyceae (e.g., *Heterococcus* sp. and *Chlorellidium* sp.), a variety of other potentially photosynthetic Ochrophyta, a diversity of predators (nematodes, rotifers, and tardigrades), and a fungus (*Geranomyces* sp.) (Figure 4B). The Ochrophyta, a phylum of Stramenopiles, which include the class Xanthophyceae (yellow-green algae), represent an exceptionally diverse group of protists with both photosynthetic and non-photosnythetic members (Cavalier-Smith and Chao, 2006; Yang et al., 2012). Much is still to be learned about this clade and the results herein suggest they are worthy of more intensive investigation in the MDV. The nematode *Plectus* and the rotifer *Adineta* are commonly found in wetter areas of the MDV which includes areas with crusts (Porazinska et al., 2002; Fontaneto et al., 2015). Consistent with the relative abundance analyses, our microinvertebrate count data (Table 2) revealed higher abundances of rotifers and *Plectus* in the moist gully and moraine while *Scottnema* was the most abundant nematode in the dry near gully (Table 2 and Supplementary Table 1). Additionally, no tardigrades were observed in the dry near gully while being present in each gully and moraine sample (Table 2). In contrast, only one phylotype, a *Colpodella* sp., showed significant differential relative abundance in the non-crust community (Figure 4B). *Colpodella* species have been previously identified in Taylor Valley soils with moderately low moisture levels of 3.1–4.9% (Fell et al., 2006) and are known as generalist predators of algae, ciliates and other protozoa (Olmo et al., 2011; Heidelberg et al., 2013; Sam-Yellowe et al., 2019). It may be that their generalist lifestyle allows them to be intermittently active during rare periods of higher soil moisture, utilizing any form of available prey.

### Do Habitats With Higher Soil Moisture Support More Diverse Communities?

Higher richness was found in the moister crust habitats (gully and moraine) than the drier non-crust habitats (near gully and slope) for both 16S (*p* < 0.003) and 18S (*p* < 0.001) (Figure 5). However, between the dry non-crust habitats, the near gully had significantly greater richness than the slope for both 16S (*p* < 0.001) and 18S (*p* < 0.001). The influence of soil moisture on microbial richness in the MDV varies depending on the valley and habitat type. For example, no correlation between sediment water content and bacterial richness was found in Wright Valley along moisture gradients perpendicular to the Onyx River (Zeglin et al., 2011). However, a correlation between community composition and sediment water content was present. Conductivity (as a proxy for salinity) was suggested as the primary factor controlling richness in those sediments. Higher salinity in Pearce Valley soils also seemed to play a role in microbial richness but not on community composition (Chan-Yam et al., 2019). In Miers Valley soils, both effects were observed with bacterial richness positively correlated with soil water content and negatively correlated with soil conductivity (Bottos et al., 2020).

### Are Phototrophs of the IMZ Also Found in Lower Elevations of the MDV or Other Cold Deserts?

Perhaps the most important finding of this study in terms of organismic diversity is the identity of both bacterial and eukaryotic phototrophs. In this regard we wanted to know if these phototrophs are endemic to the IMZ or if they are similar to phylotypes found elsewhere in Taylor Valley and other cold deserts. The most conspicuous phototroph was a moss that dominated (in relative abundance) the crusts of the gully (79% of sequences) and the moraine (32%) but not the non-crust communities (Figure 7). This moss also had the greatest difference in relative abundance for all eukaryotes between crust and non-crust communities (Figure 4B). The identity of the moss was the same in both the gully and moraine habitats (ESV 5). The 100% match of the moss to a putative *Bryum* species (NCBI acc # KT343959) from Botany Bay on the nearby coast (Seppelt et al., 2010), and to an environmental sequence (acc # HM490232) from hypolithic communities in the Miers Valley (Khan et al., 2011) suggests it is likely not endemic to the IMZ but may be endemic to Antarctica. Although it is not possible to determine the exact species identity of this moss using only short-read 18S sequences, this finding points to future work using a variety of genes to examine the biogeography of this and related mosses in Antarctica and across the cryobiosphere.

Like the moss, other abundant eukaryotic phototrophs in the crust communities also showed high 18S sequences similarity to previously studied phototrophs in Antarctica and other cold ecosystems. For example, an abundant phylotype in the gully and moraine (ESV 1) is a *Chlorococcum* sp. (formerly *Pleurastrum*, Kawasaki et al., 2015) that shares 100% identity with isolates from several sites in Antarctica (De Wever et al., 2009), including two isolates (acc# FJ946903, FJ946905) from Lake Fryxell and an isolate (acc# FJ946902) from Ace Lake (over 2,500 km from the Dry Valleys). At an even broader geographic scale, it shares over 99% identity with many environmental sequences from peri-glacial sediments in the high Himalayas (e.g., HQ188976, Schmidt et al., 2011) and from the debris-covered Toklat Glacier in central Alaska (e.g., KM870774, Schmidt and Darcy, 2015) indicating that this group (formerly the “CP-clade”) has broad geographical distribution in the cryosphere as discussed previously by Schmidt et al. (2011) and Sommers et al. (2019a). Given that phylotypes in this group have been isolated from Antarctic lakes, cryoconite holes, and peri-glacial soils throughout the world, it is currently unclear if they are functional components of the terrestrial sites in the Dry Valleys, or if they are dormant organisms deposited there from nearby lakes such as Lake Fryxell. Given that they are more abundant in wetter sites in the present study and that they dominate soils that are not near lakes in the Himalayas and Alaska, it is likely that they are functioning components of soil crust communities in the Dry Valleys, but much more work is needed on these abundant soil algae.

Despite differences in relative abundance of eukaryotic phototrophs between crust and non-crust habitats, it is important to note that phototrophic phylotypes were among the top 10 18S phylotypes in all habitats. For example, the dry near gully had a relatively abundant phylotype (ESV 25) in the Trebouxiophyceae (Figure 7) which was 100% identical to sequences (e.g., acc.# LC487925) of lithic crustose lichen photobionts in Queen Maud Land, Antarctica and other locations (Sweden, acc.# MH807084 and Germany, acc. # MH807084). Though lichen diversity in Taylor Valley is comparable to other ice-free areas of the Ross Sea region (Colesie et al., 2014); they exist in a narrow zone with regards to snow accumulation. Too much or too little snow and they are not able to survive (Green et al., 2012) and are generally found only in higher elevations of the IMZ. The dry slope also had a potential phototroph (ESV 2) in its top 10, a diatom of the Ochrophyta phylum of Stramenopiles that are incredibly diverse (Raven and Giordano, 2017; Varela et al., 2017), including some non-photosynthetic species (Kamikawa et al., 2017).

In contrast to the eukaryotic phototrophs, bacterial phototrophs were more restricted to the wetter crust habitats (Figure 6) similar to patterns observed in soils near MDV lakes, ponds, and streams (Zeglin et al., 2009; Niederberger et al., 2015; Van Horn et al., 2016). *Nostoc* phylotypes were the most and second most abundant ESVs in the gully and moraine habitats, respectively (Figure 6) agreeing with previous studies showing that Nostocales are found almost exclusively in moister soils of the Taylor Valley (Buelow et al., 2016) where they are likely important nitrogen fixers (Coyne et al., 2020). The most abundant phylotype in the gully (ESV_203) was a 100% match across our relatively short reads (253 bp) to several *Nostoc* sequences, including one (accession # MN243125, Jung et al., 2019) from a biological soil crust in the high Arctic (Ny-Alesund, Spitsbergen). Longer sequence reads and a more polyphasic approach would be needed to assess the biogeography of these and other phototrophs discussed here, but these results have revealed interesting organisms for future in-depth study. Likewise, the most abundant phylotype on the moraine (ESV_44) was a *Phormidium* with a 100% match for many sequences from mats of Lake Fryxell down valley from our study sites (e.g., AY151763, Taton et al., 2003), and a 99.2% match to sequences from a high-elevation site in the Himalayas (e.g., HQ188993, Schmidt et al., 2011). Also, in the top 10 phylotypes of the moraine was a strain of *Phormidesmis* sp. (ESV 736), a genus known to be associated with glacial environments and microbial mats near both poles (Raabova et al., 2019). Given its ability to produce exopolysaccharides (EPS), it is well suited for living under polar conditions (Christmas et al., 2016).

As mentioned above, the drier, non-crust sites of the near gully and slope were mostly dominated by members of the Actinobacteria and Acidobacteria (Figure 6). Actinobacteria are common in dry soils in the MDV regardless of geomorphic zone (Pointing et al., 2009; Lee et al., 2012; Van Goethem et al., 2016) and are ubiquitous in cold deserts across the Earth (An et al., 2013; Gupta et al., 2015). The most abundant actinobacterium belonged to the genus *Crossiella* that has been identified in Saharan dust in the Alps and may be snowpack colonizers (Chuvochina et al., 2011). While Actino- and Acidobacteria generally dominated the dry soils, the most abundant bacterium in the near gully was *Segetibacter* sp., a member of the Bacteroidetes. *Segetibacter* spp. have been found in soils elsewhere on the continent (Kudinova et al., 2020) and in high elevations throughout the world (Solon et al., 2018; Vimercati et al., 2019a; Aszalós et al., 2020) and are found in snowpack (Maccario et al., 2019).

Among the most abundant Acidobacteria in both the near gully and slope was *Blastocatella* spp. One of the more abundant *Blastocatella* phylotypes (ESV 47), however, was also present in moist soils of the moraine. Phylotypes similar to *Blastocatella* were previously reported from other high elevation ecosystems, including lake sediments from Ojos Del Salado in the Andes (acc.# LN929609, Aszalós et al., 2020).

The most cosmopolitan genus across all four habitats was *Sphingomonas* (Alphaproteobacteria). Previous studies in the MDV have also uncovered *Sphingomonas* species in a variety of habitats ranging from the mats and moats of lakes (Brambilla et al., 2001; Van Trappen et al., 2002) to lithic environments (Gunnigle et al., 2015). A strain of *Sphingomonas sediminicola* collected from tundra soil on Svalbard in the Arctic circle (acc.# MH929654) provided the closest match (100%) to sequences of *Sphingomonas* species in our soils. The genus is known for being cosmopolitan and its EPS production and biodegradative abilities make it ecologically versatile (Baraniecki et al., 2002; Asaf et al., 2020) perhaps explaining its widespread occurrence in our study.

### Broader Relevance of This Study

The initial impetus for this study was to explore the mountain slopes above Canada Glacier for possible sources of inoculum for cryoconite holes on Canada Glacier and other glaciers in the MDV. Many of the more abundant cyanobacterial genera found in the crust communities (e.g., *Nostoc*, *Phormidium, Phormidesmis*) were also found to be abundant in cryoconite holes on glaciers of Taylor Valley (Porazinska et al., 2004; Sommers et al., 2019a). Likewise, *Chlorococcum* sp., the most abundant algal phylotype in crusts was also the most common alga in cryoconite holes of the valley (Sommers et al., 2019a). This link is also seen with heterotrophic bacterial genera common in our soils (e.g., *Sphingomonas*, *Blastocatella*) that were also found in high relative abundance in cryoconite holes across Taylor Valley (Sommers et al., 2020). Although an in-depth analysis of the comparisons between terrestrial mountain habitats and cryoconite hole sediments is beyond the scope of this paper, more investigation into whether high-elevation soils are the main source of inoculum for cryoconite holes is warranted.

## Conclusion

The data presented here indicate the gully and moraine habitats are acting as islands of biodiversity in this dry, windswept environment. Both habitats accumulate excess snow compared to the surrounding landscape (see Supplementary Figures 1, 3, 4) and had higher soil water content (Figure 2). Positive effects of increased snow accumulation on soil water content and microinvertebrate population size has been documented in lower elevations of the MDV (Gooseff et al., 2003; Ayres et al., 2010) and studies of other cold, dry environments show topographic features that promote snow accumulation (i.e., depressions and ridgelines) serve as hotspots of microbial diversity and functioning across otherwise barren high-elevation landscapes (e.g., Costello et al., 2009; Solon et al., 2018; Vimercati et al., 2019b). A similar effect is also seen in hot deserts where increased water accumulation strongly influences microbial and crust diversity (Kidron et al., 2000; Kidron and Benenson, 2014; Hagemann et al., 2017). However, to our knowledge there has been no previous work examining the microbial diversity of gullies in the MDV despite recent attention focused on these landscape features by geomorphologists (Dickson and Head, 2009; Levy et al., 2009; Dickson et al., 2019). Likewise, lateral and medial moraines have received very little attention as possible oases for life (Mapelli et al., 2011; Darcy et al., 2017) or as sources of cryoconite (Franzetti et al., 2017), but the present study indicates that they may be important hotspots of microbial diversity and activity in glacial and periglacial environments. Obviously, much more work is needed to fully understand how topographic features lead to enhanced microbial activity and functioning across extreme high-elevation landscapes.

## Data Availability Statement

The data presented in the study are deposited in the NCBI BioProject database under accession number PRJNA721735. The data is publicly available at the following link: https://www.ncbi.nlm.nih.gov/bioproject/721735.

## Author Contributions

SS acquired funding for the study. AS, PS, LV, JD, DP, and SS contributed to the design of the study and conducted the field sampling. DP processed and counted the microinvertebrates. EG, LV, and CM processed samples for sequencing and gravimetric soil moisture. AS, CM, and EG performed data analysis. AS wrote the first draft of the manuscript. SS wrote sections of the manuscript. AS and SS contributed equally to the final manuscript. All authors contributed to manuscript revision and approved the submitted version.

## Conflict of Interest

The authors declare that the research was conducted in the absence of any commercial or financial relationships that could be construed as a potential conflict of interest.

## Footnotes

1 https://inkscape.org/

2 https://github.com/fiererlab/dada2_fiererlab

3 http://benjjneb.github.io/dada2/tutorial.html
