# Supplementary material for: Gullies and Moraines Are Islands of Biodiversity in an Arid, Mountain Landscape, Asgard Range, Antarctica
Source: Front Microbiol. 2021 Jun 10;12:654135. doi: 10.3389/fmicb.2021.654135 (PMC8222675; doi:10.3389/fmicb.2021.654135)
Supplement: Supplementary file 2 [file Data_Sheet_1.docx]

Supplementary Materials 1
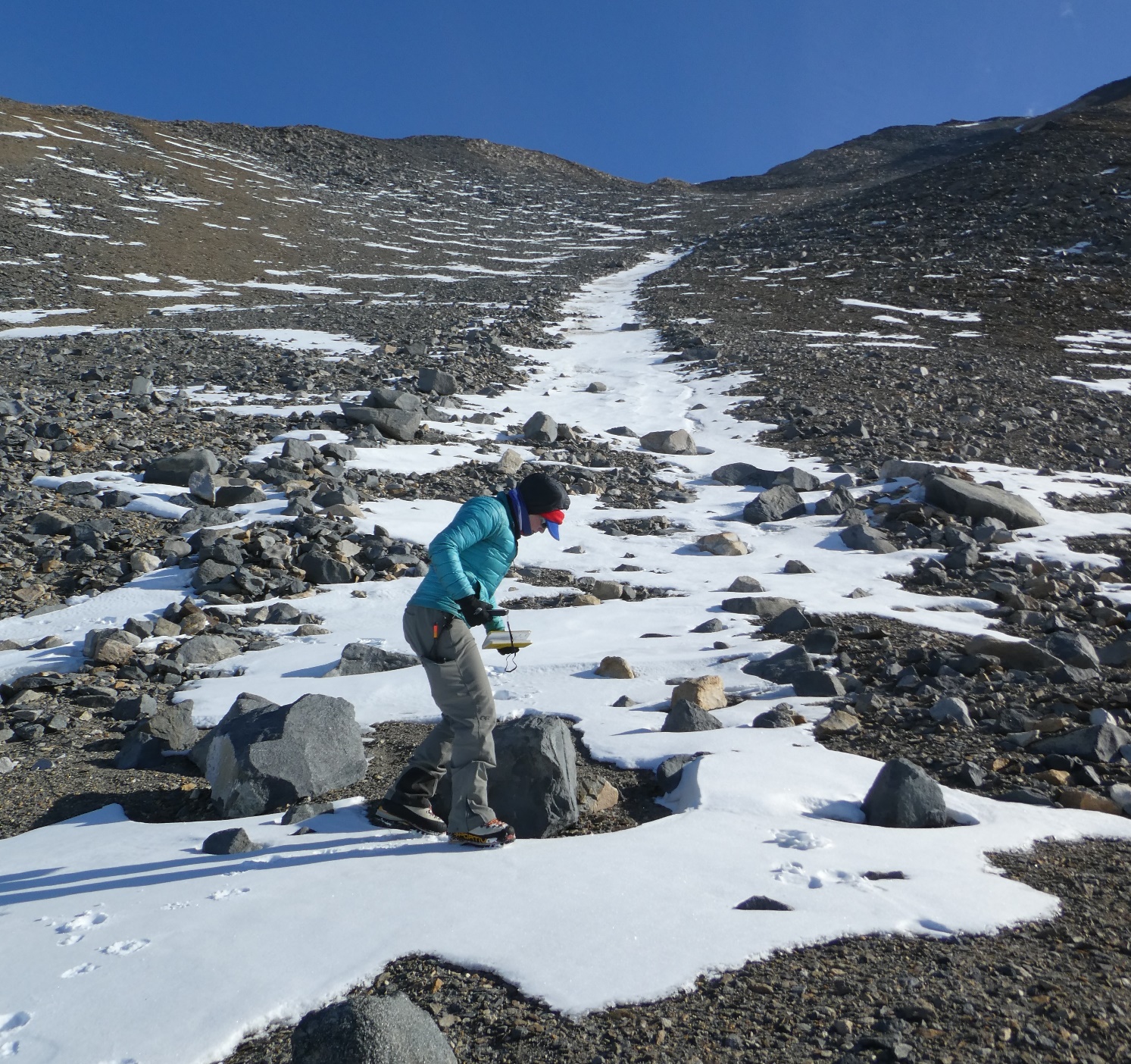


Supplementary Figure 1. View of the lower reach of the Gully 20 Nov 2018 looking uphill from the first sampling point, illustrating how the Gully accumulates snow. For this paper the gully was sampled during the prior summer (22 Jan 2018) when it was completely free of snow. Pacifica Sommers shown for scale. Photo: Adam Solon

**
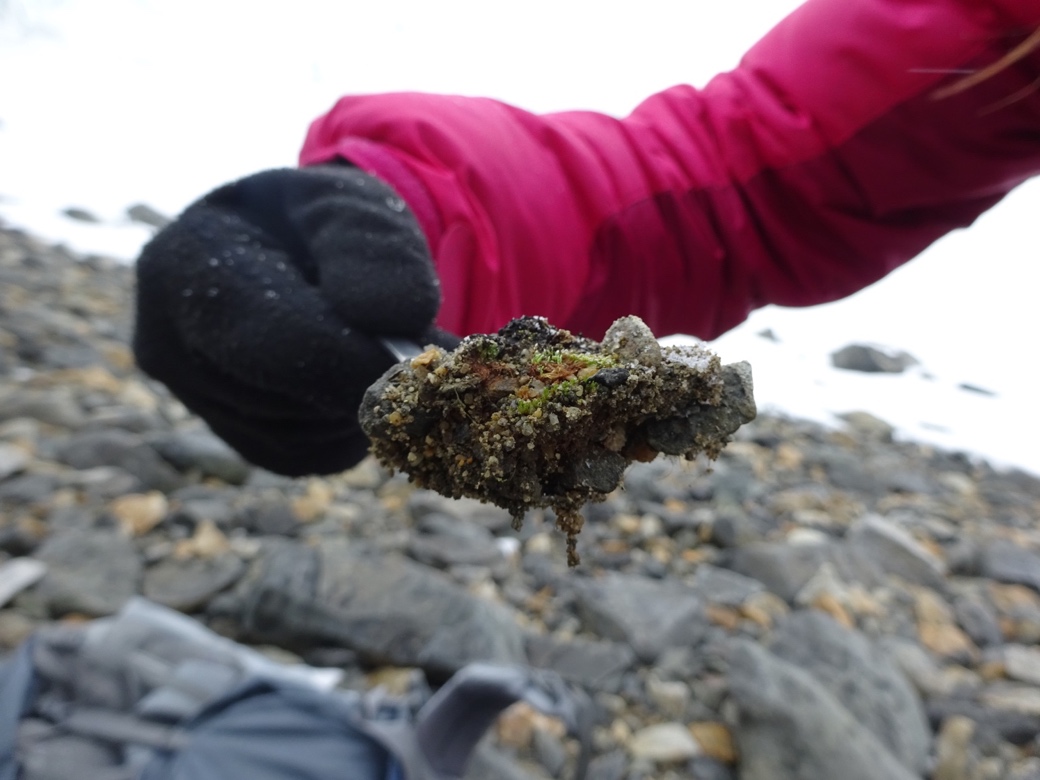
**

**
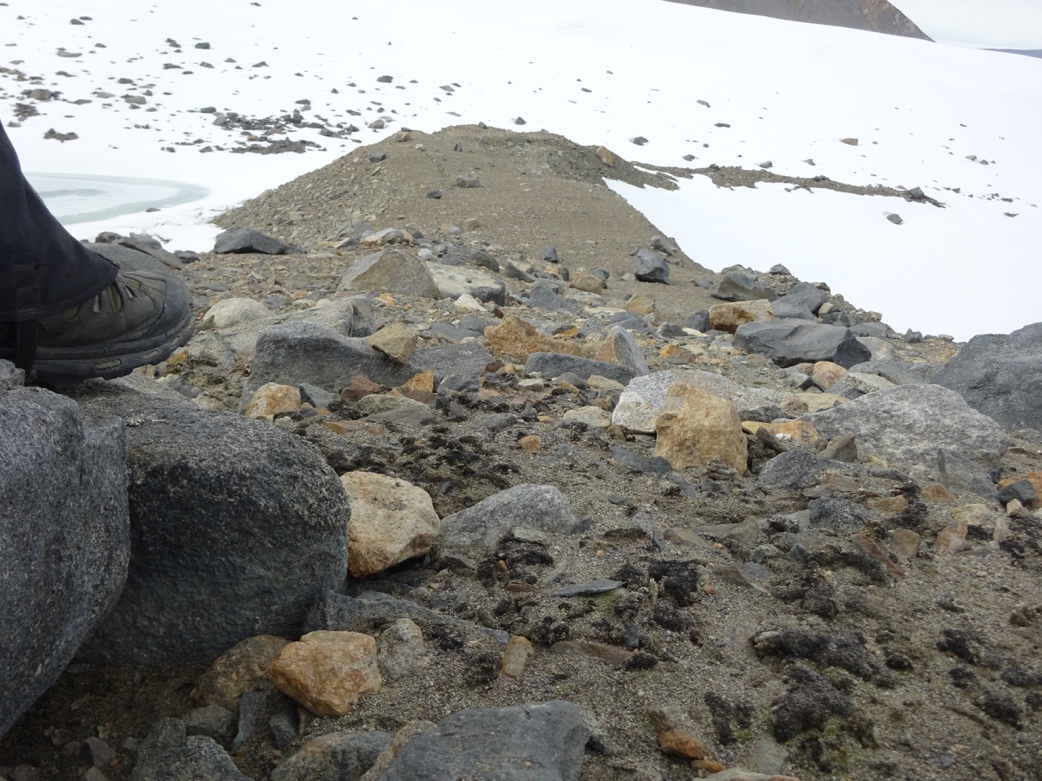
**

**Supplementary Figure 2.** Top photo. Sample 5 (in table spoon for scale) from the Moraine showing moss and black crust. Developed crust such as this were found only in the lower 4 sites on the Moraine. The upper reaches of the Moraine did not have developed crusts. Photo: S.K. Schmidt (25 Jan 2018).

Bottom Photo. Black crust at the bottom of the Gully near the “dam”. The pond and Moraine are visible in the upper left of the photo. Photo: S.K. Schmidt (22 Jan 2018).

**
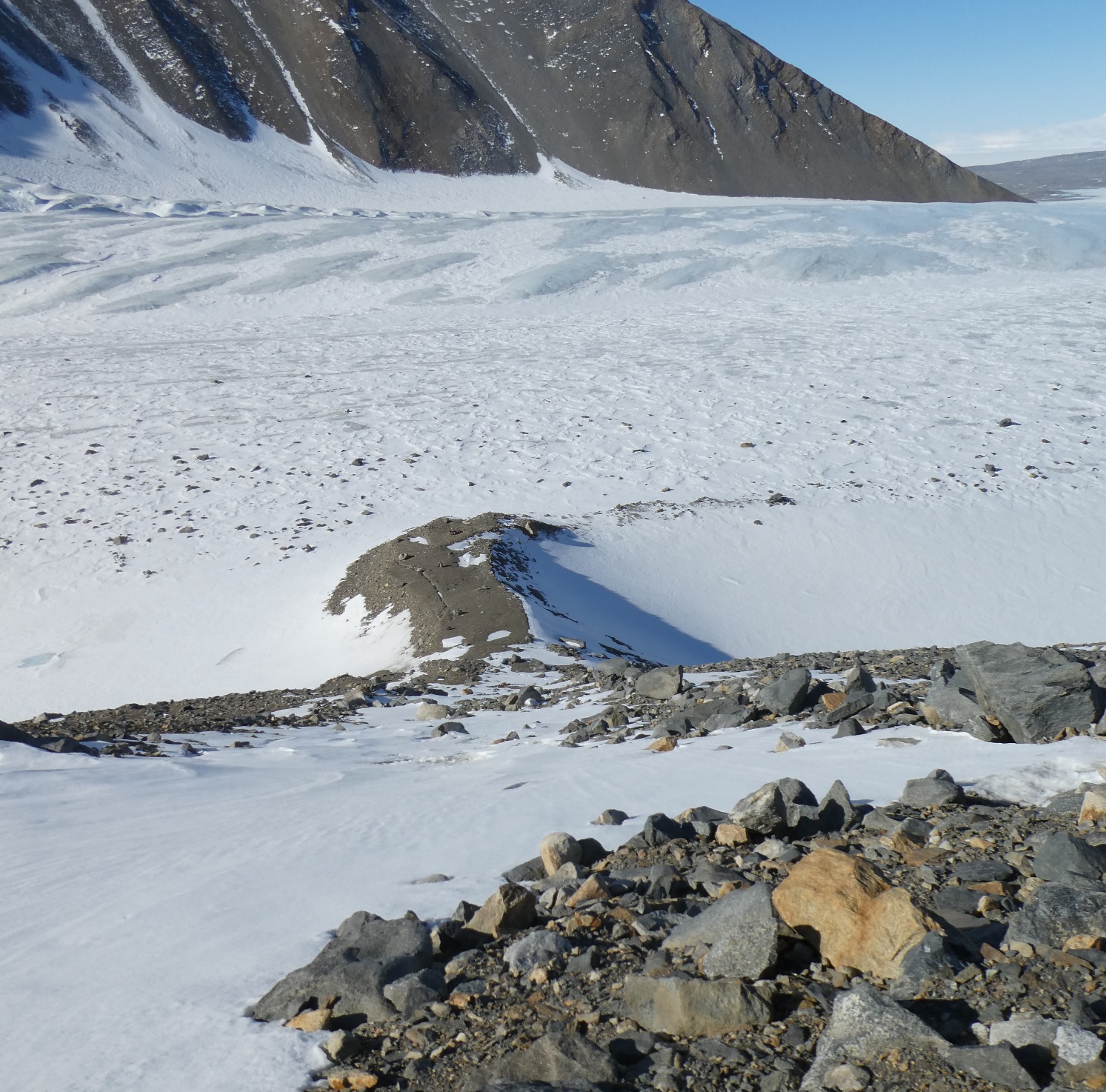
**

**Supplementary Figure 3.** View of the Gully habitat looking down toward Canada Glacier from the last sampling point. Note the dam that connects the Gully to the Moraine or the glacier. Photo: Adam Solon (20 Nov 2018)

**
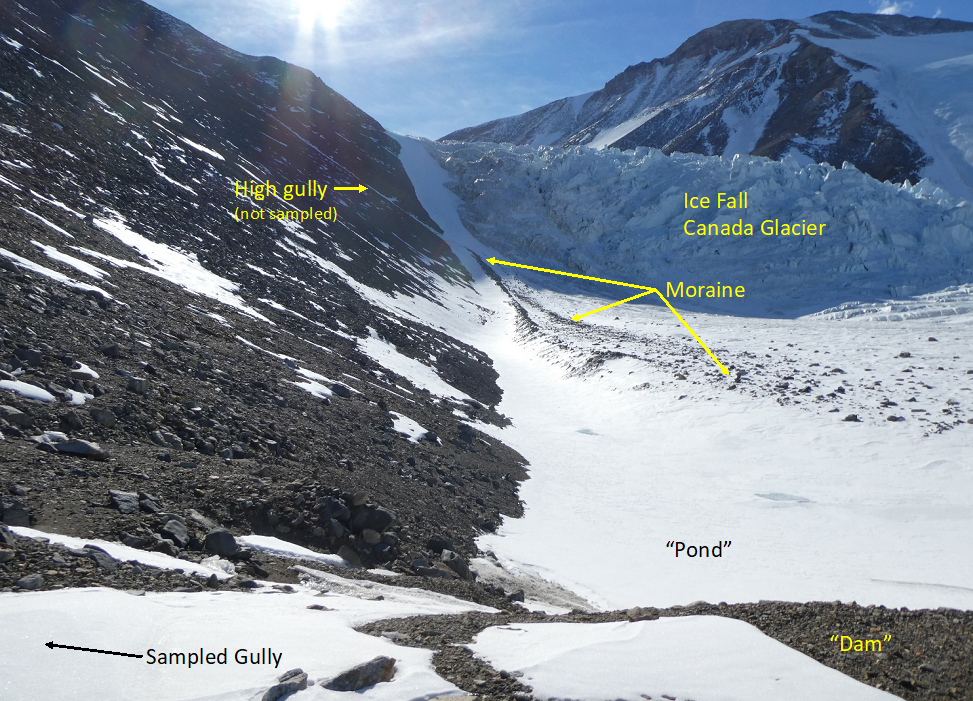
Supplementary Figure 4**. View of the Moraine photographed from the “dam” at the bottom of the Gully (20 NOV 2018). The highest elevation sample taken on the Moraine was where the “High gully” feeds sediments onto the Moraine. The photo also shows accumulation of snow on the Moraine. Photo: Adam Solon

**Supplementary Figure 5.** View of the dam and Gully from the top sampling site on the Moraine where the high gully feeds onto the Moraine. Photo was taken on the date the Moraine was sampled (25 Jan 2018). Photo: S.K. Schmidt.
