# Supplementary material for: Gullies and Moraines Are Islands of Biodiversity in an Arid, Mountain Landscape, Asgard Range, Antarctica
Source: Front Microbiol. 2021 Jun 10;12:654135. doi: 10.3389/fmicb.2021.654135 (PMC8222675; doi:10.3389/fmicb.2021.654135)
Supplement: Supplementary file 3 [file Data_Sheet_2.docx]

Supplementary Material 2


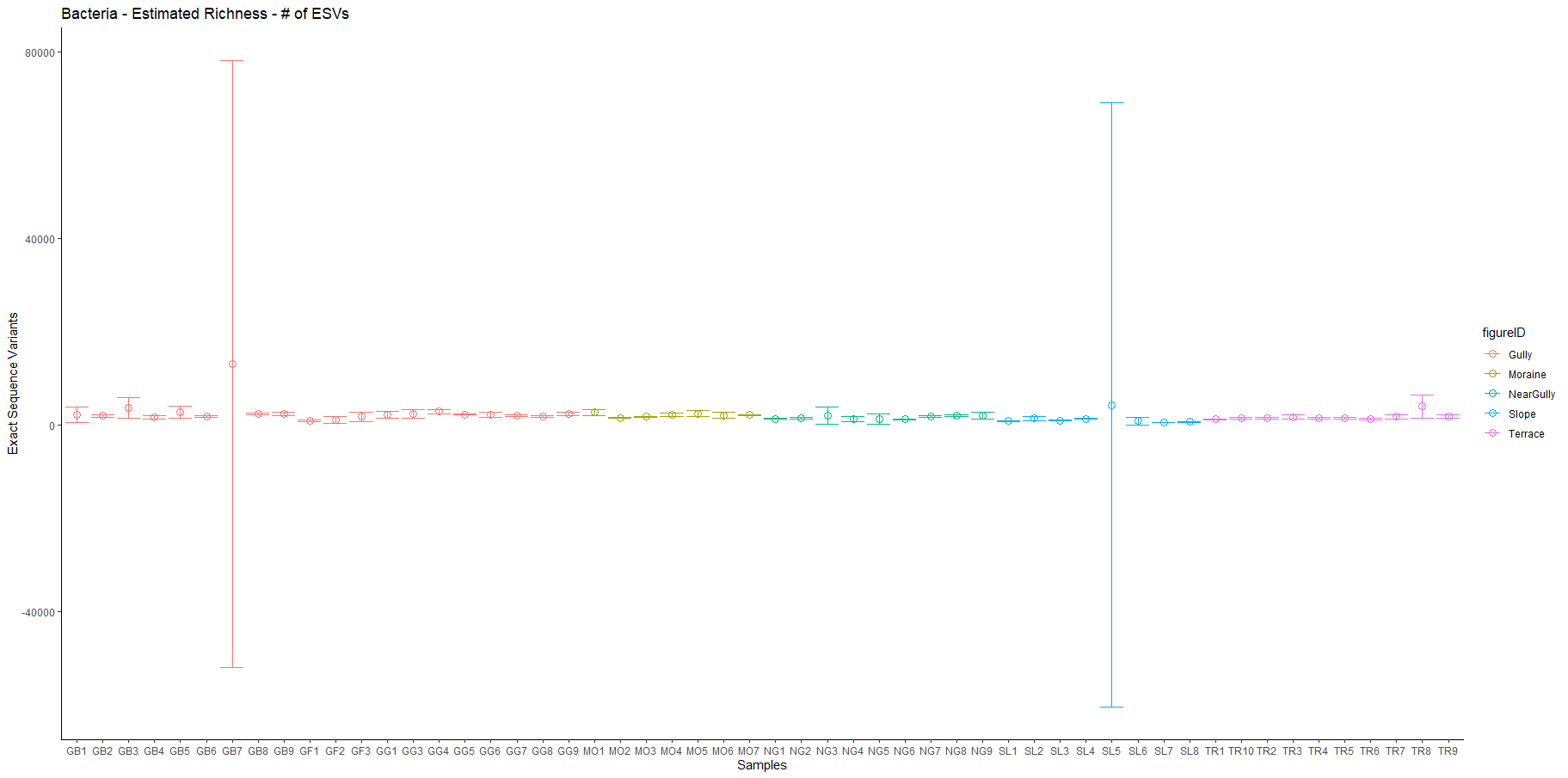


Supplementary Figure 1. Bacterial and archaeal richness estimates w/ standard errors for each sample. Samples with unusually large error bars that include negative numbers of ESVs (which is not biologically possible)- GB7 (left) and SL5 (right)- were removed for the richness estimation and null hypothesis significance testing used in the manuscript.
